# Evaluation of the additive effect of interferon α 2b with monthly intravitreal injection of bevacizumab in refractory diabetic macular edema

**DOI:** 10.1186/s40942-022-00424-x

**Published:** 2022-10-12

**Authors:** Hooshang Faghihi, Bahman Inanloo, Arash Mirzaee, Kaveh Fadakar, Ahmad Mirshahi, Nazanin Ebrahimiadib, Fariba Ghassemi, Fatemeh Bazvand, Abdulrahman Amini, Masoud Mirghorbani, Shahin Faghihi, Elias Khalili Pour, Hamid Riazi-Esfahani

**Affiliations:** Retina Service, Farabi Eye Hospital, Tehran University Of Medical Sciences, Qazvin Square, South Karegar Street, Tehran, 1336616351 Iran

**Keywords:** Diabetic retinopathy, Refractory diabetic macular edema, Interferon-alpha, Anti-vascular endothelial growth factor (Anti-VEGF)

## Abstract

**Background:**

To evaluate the additive effect of topical or sub-tenon injection of interferon (IFN)-α 2b in the treatment of refractory diabetic macular edema.

**Methods:**

In this prospective study patients with center-involved DME who were unresponsive to 3 monthly consecutive IVB injections were recruited. Patients were divided into three groups: group1, received IFN- α 2b topical drop at a dose of 1mIU/ml four times a day for 3 months. Group 2, received a single sub-tenon injection of 1mIU/ml IFN- α 2b at the enrollment. Group 3 received artificial tears four times a day for 3 months (control group). All groups received three consecutive monthly IVB injections and were evaluated monthly up to 1 month following the last IVB injection.

**Results:**

In this study, 59 eyes of 35 patients with refractory DME were assessed. The final follow-up showed that although CMT decreased in all groups, only patients in Group 2 had statistically significant lower CMT compared to their baseline values (change in CMT: − 117 ± 213 µm; p-value = 0.025). Comparison of CMT changes between three groups showed no statistically significant difference, although it was higher in group 2 (change in CMT: − 117 ± 213 µm (Group2) vs. − 49 ± 173 (Group 1) vs. − 36 ± 86 (Group 3); p-value = 0.085). Considering eyes with baseline CMT > 400 µm, sub-tenon injection of IFN α2b led to a significant reduction of CMT at the first month and final follow-up visit (CMT change: − 166 ± 210, − 145 ± 231 µm; p-value = 0.018 and 0.035, respectively). In this subgroup, eyes in Group 2 had lower CMT at the first month following treatment in comparison with the control group (CMT: 444 ± 123 µm vs. 544 ± 96 µm, p-value = 0.042). Alterations of CDVA were not statistically significant among groups, although patients in Group 1 had a significant improvement in vision at second and last follow up (CDVA change: − 0.23 ± 0.39, − 0.20 ± 0.43 logMAR; p-value = 0.030 and 0.010, respectively).

**Conclusions:**

In short term, Sub-tenon injection of IFN might have an additive anatomical effect in eyes with refractory DME. Validation of this observation requires further prospective controlled studies.

## Background

Diabetic macular edema (DME) is the leading cause of blindness in patients with diabetic retinopathy [1]. During a 10- to 20-year follow-up, DME affects 20–40% of diabetic individuals [2]. Persistent DME causes damage to the photoreceptors and leads to permanent vision loss therefore vigilant treatment is crucial to prevent vision reduction [3]. Vascular endothelial growth factor (VEGF) is a pivotal mediator of blood-retinal barrier breakdown that leads to the development of DME [4]. Based on the increased level of intraocular VEGF in DME, injection of anti-vascular endothelial growth factor (anti-VEGF) is the first-line treatment of center-involved DME (CI-DME) [5]. Anti-VEGF agents such as Pegaptanib, Ranibizumab, Bevacizumab, and Aflibercept have been shown to be effective for the management of DME [6, 7]. Among them, intravitreal Bevacizumab (IVB) is used widely due to its cost-effectiveness and safety for the treatment of DME especially in developing countries [8, 9].

Despite the positive effects of IVB in these patients, DME can be refractory in some cases. As a result, alternative anti-VEGF drugs, argon macular lasers, intravitreal steroid injections, and nonsteroidal anti-inflammatory drugs (NSAIDs) have all been proposed as alternative treatment options [10]. Additionally, some investigators advocated interferon(IFN)-α2b in DME [11, 12]. Interferons are glycoproteins that are secreted in response to foreign pathogens and tumor cells. They are a subset of a larger group of biological molecules known as cytokines. Interferons have antiviral, immunomodulatory, and proliferation inhibitory properties, as well as the ability to inhibit other signaling pathways such as VEGF, interleukin-8, interleukin-10, transforming growth factor-beta, and tumor necrosis factor-α [13].

So far, IFN-α has been used in various ophthalmologic diseases with a good safety profile [14]. In cases where interferon is used systemically, reported side effects were muscle pain, weight loss, flu-like symptoms, hypotension, tachycardia, drowsiness, retinal involvement as retinal hemorrhage, ischemia, and nerve fiber layer defects [12]. Topical IFN-α2b is the principal treatment for ocular surface squamous neoplasia (OSSN), and no substantial systemic or ocular complications have been documented, other than mild eye surface irritation and conjunctival hyperemia [15–17]. It was shown that the IFN α level in aqueous is significantly lower in diabetic patients compared with non-diabetics [18]. Also, an in vitro improvement of barrier function of bovine retinal endothelial cell was reported previously [19]. Although the precise mechanism of action, drug penetration of topical IFN in the posterior region, and resolution of macular edema are not fully investigated, topical IFN- α2b therapy has been used successfully in the treatment of pseudophakic macular edema as well as uveitic maculae edema [20, 21]. A randomized controlled trial (RCT) has also demonstrated the safety and some beneficial effects of topical IFN-α in patients with diabetic macular edema (DME) [12].

Sub-tenon injections are another route for intraocular drug delivery [22]. The main advantage of sub-tenon injection of drugs such as corticosteroids over other routes is that it can deliver large amounts of the drug to the eye over a longer period of time [22, 23]. Sub-tenon injection improves drug availability and reduces the risk of side effects [22]. The safety and efficacy of IFN-α sub-tenon injections have been evaluated in some previous studies [24, 25].

This study aimed to assess the additive effect of administering topical or sub-tenon injections of IFN- α2b in the treatment of DME resistant to three consecutive IVB injections, taking into account the probable anti-angiogenic and anti-inflammatory effects of IFN-α, its safety profile, and the lack of evidence for application of IFN in refractory DME.

## Methods

This prospective comparative interventional case series was conducted in Retina Clinic of Farabi Eye Hospital, Tehran University of Medical Sciences, from April 2020 to March 2021. The study protocol was approved by Tehran University of Medical science’s Institutional Review Board (IR.TUMS.FARABIH.REC.1400.061) and adhered to the principles outlined in the Declaration of Helsinki. All participants were thoroughly explained about the protocol of the study and written informed consent was obtained from each patient before entry into the study.

Diabetic patients (type 1 or 2) with CI-DME who had Central macular thickness (CMT) ≥ 300 microns in Optical Coherence Tomography (OCT) and showed less than 50 µm or 10% reduction of CMT after three consecutive monthly injections of IVB were recruited in this study. Patients with uncontrolled glaucoma, uncontrolled diabetes (HbA1c > 10), systolic blood pressure > 160 mmHg, intraocular surgery in the last 6 months, active proliferative diabetic retinopathy, significant disease in the vitreomacular interface, intravitreal or periocular steroid injection in the last 6 months, and argon laser treatment in the retina in the last 3 months were excluded. Additionally, monocular patients and those who may require vitreoretinal surgery in the future were excluded (such as epiretinal membrane or tractional retinal detachment). Before enrollment, all patients had at least three consecutive monthly intravitreal injections of 1.25 mg/0.05 ml bevacizumab, and enrollment occurred between 4 and 6 weeks after the last IVB injection.

Baseline characteristics of patients including age, sex, concomitant systemic diseases, and HbA1C were recorded. Corrected distant visual acuity (CDVA) using Snellen chart and refraction were assessed. All visual assessments were performed in a single center by a group of expert optometrists using the identical lighting, distance, and chart circumstances. All participants underwent thorough ophthalmological examinations including slit-lamp biomicroscopy, dilated fundoscopic exam. Intraocular pressure determined using Goldmann Tonometer. CMT was measured for all eyes with OCT Spectral-domain optical coherence tomography (Spectralis, Heidelberg, Germany). All assessments were performed in the morning to prevent the effect of diurnal variations.

Eligible patients were divided into three groups blindly based on numerical order. Group 1: patients received IFN-α2b topical drop at a dose of 1mIU/ml four times a day for 3 months. The optimum dosage of the topical IFN-α2b was determined based on a published randomized clinical trial on the treatment of macular edema of patients with diabetic retinopathy and also available literature on the safety of treatment of ocular surface squamous neoplasia [12, 16]. A topical IFN-α2b 1mIU/ml was prepared by mixing a full vial of IFN- α2b 3mIU/ml (PDferon-B; Pooyesh DarouCo, Tehran, Iran) with 2 ml of balanced saline solution. The drug was prepared weekly and delivered to the patients. The patients were instructed to store the medication in the refrigerator at 4 °C. Group 2 patients received a single sub-tenon injection of IFN-α2b at a dose of 1mIU/ml at the enrollment. The dose of 1mIU/ml of sub-tenon injection has been evaluated in previous studies by Cellini et al. [24, 25]. After the administration of topical anaesthesia, 1 ml of IFNα was slowly injected into the inferotemporal quadrant under the Tenon’s capsule, using a 27-gauge needle on a 1-ml syringe. The needle was moved toward the macular area, until the hub was firmly pressed against the conjunctival fornix. Group 3 received artificial tears four times a day for 3 months and considered as control group. All groups received 3 monthly consecutive intravitreal injection of bevacizumab 1.25 µg/0.05 ml in operating room (OR) during the study period. Therefore, in patients with bilateral refractory DME, both eyes received a same protocol.

Patients were visited monthly until 1 month following their final IVB injection, and CDVA and CMT values were measured at each visit. Throughout the trial period, participants were monitored for adverse effects of IFN-α. In the event of serious ocular or systemic complications, treatment was discontinued.

### Statistical analysis

Descriptive statistics such as frequency, percentage, mean, and standard deviation were used to describe the data. The normal distribution of quantitative variables was assessed by the Kolmogorov–Smirnov test. Comparison between the groups at baseline was accomplished by the ANOVA test, Kruskall-Wallis, and Chi-square test. To analyze the alteration within each group during the follow-up visit a linear mixed model as well as Wilcoxon Ranked test was applied. To compare changes of variables among groups during follow-up analysis of covariance (ANCOVA) was used to adjust for the baseline values. p-value < 0.05 indicated the statistical significance using SPSS software (IBM SPSS Statistics for Windows, Version 24.0; IBM Corp., Armonk, NY).

## Results

In this study, 23 eyes from 14 patients, 16 eyes from 10 patients, and 20 eyes from 11 patients were enrolled in group 1, 2 and 3, respectively. At baseline, there was no significant difference between groups regarding age, spherical equivalence, pseudophakic lens status, HbA1C, Intraocular pressure (IOP), CMT, and CDVA (p-value > 0.05). Table 1 shows the baseline characteristics of the participants in the groups.

**Table 1:** Baseline characteristics of the participants in three groups

Variables	Group 1 (IVB + IFN Drop) N = 14 (23 eyes)	Group 2 (IVB + IFN Injection) N = 10 (16 eyes)	Group 3 (IVB) N = 11(20 eyes)	p-value
Age (years)	63 ± 8	62 ± 9	59 ± 6	0.352
Sex number (%)				
Female	13 (56%)	9 (57%)	3 (15%)	**0.007**
Male	10 (34%)	7 (43%)	17 (85%)	
Spherical Equivalence (diopter) mean ± SD	0.64 ± 1.15	0.89 ± 1.85	0.84 ± 1.5	0.866
Lens Status number (%)				
Pseudophakic	10 (43%)	7 (43%)	6(30%)	0.597
Phakic	13(56%)	9 (57%)	14(70%)	
HbA1C (%) mean ± SD	7.73 ± 1.01	8.04 ± 0.64	7.69 ± 0.72	0.370
IOP (mmHg) mean ± SD	17 ± 4.3	16 ± 4	17 ± 2.5	0.592
CMT (microns) mean ± SD	566 ± 202	560 ± 238	543 ± 179	0.923
CDVA (logMAR) mean ± SD	0.91 ± 0.56	0.71 ± 0.43	0.76 ± 0.49	0.529

Bold values denote statistical significance at the p-value < 0.05 level

IVB, intravitreal bevacizumab; HbA1C, glycated hemoglobin; IOP, intraocular pressure; CMT, central macular thickness; CDVA corrected distal visual acuity

Table 2 demonstrates the alteration of CMT and CDVA for each group during follow-up. All three groups showed a reduction of CMT during follow-ups. Comparison of CMT at each time point revealed a significant decrease following treatment at the second month for IFN drop (Group 1) and IVB (group 3) monotherapy group (p-value = 0.006 for the both groups). Although the CMT at the third month is still lower than the baseline value in these two groups, the difference is not statistically significant. However, eyes receiving sub-tenon IFN injection (Group 2) had significantly lower CMT than the baseline value at third month (443 ± 128 µm vs 560 ± 238 µm, p-value = 0.025).

**Table 2:** Mean CMT and CDVA of three groups at baseline and follow up visits

Variables		Groups			Total P-value	P-value (Group 1 vs. 2)	P-value (Group 1 vs. 3)	P-value (Group 2 vs. 3)
		IVB + IFN Drop (Group 1)	IVB + IFN Injection (Group 2)	IVB (Group 3)				
CMT (microns)	Baseline	566 ± 202	560 ± 238	543 ± 179	0.907^†^	0.995	0.960	0.999
	1st month	499 ± 217	420 ± 122	494 ± 122	0.068*	0.137	0.999	0.104
	Change	− 67 ± 157	− 140 ± 194	− 49 ± 104				
	P-within^§^	0.073	**0.020**	**0.030**				
	2nd month	461 ± 166	482 ± 171	489 ± 120	0.316*	0.925	0.409	0.999
	Change	− 105 ± 151	− 78 ± 115	− 54 ± 90				
	P-within^§^	**0.006**	0.091	**0.006**				
	3rd month	517 ± 214	443 ± 128	507 ± 128	0.085*	0.145	0.999	0.151
	Change	− 49 ± 173	− 117 ± 213	− 36 ± 86				
	P-within^§^	0.239	**0.025**	0.054				
CDVA (LogMAR)	Baseline CDVA	0.91 ± 0.56	0.71 ± 0.43	0.76 ± 0.49	0.671^†^	0.815	0.822	0.999
	1st month	0.80 ± 0.49	0.61 ± 0.31	0.69 ± 0.32	0.819*	0.488	0.805	0.886
	Change	− 0.11 ± 0.41	− 0.10 ± 0.39	− 0.07 ± 0.26				
	P-within^‡^	0.145	0.260	0.216				
	2nd month	0.68 ± 0.40	0.76 ± 0.41	0.76 ± 0.41	0.739*	0.898	0.872	0.999
	Change	− 0.23 ± 0.39	0.05 ± 0.26	0.01 ± 0.18				
	P-within^‡^	**0.003**	0.496	0.802				
	3rd month	0.71 ± 0.46	0.63 ± 0.37	0.75 ± 0.38	0.769*	0.956	0.994	0.850
	Change	− 0.20 ± 0.43	− 0.08 ± 0.25	− 0.01 ± 0.13				
	P-within^‡^	**0.010**	0.308	0.723				

Bold values denote statistical significance at the p value < 0.05 level

^†^Based on ANOVA, between-group comparison adjusted by Bonferroni method

^§^Based on linear mixed model, comparison with baseline value, adjusted for the multiple comparisons by Bonferroni method

^*^Based on ANCOVA, adjusted for the baseline value, between-group comparison adjusted by Bonferroni method

^‡^ Based on Wilcoxon signed rank test, compared with baseline value

Alteration of CDVA was significant in eyes receiving IFN drop (Group 1). CDVA significantly improved in this group at the second and final follow-up in comparison to the baseline (0.91 ± 0.56 vs 0.68 ± 0.40 at the second month and vs 0.71 ± 0.46 logMAR at the final follow-up; p-value = 0.003 and 0.010, respectively).

As Table 2 shows, there was no significant difference between groups in terms of mean baseline, first month, second month, and third month’s CMT and CDVA (p-value > 0.05). Subgroup analysis comparing first versus second, first versus third, and second versus third groups regarding mean CMT and CDVA revealed no significant difference between groups as well (p-value > 0.05).

**Fig. 1:**
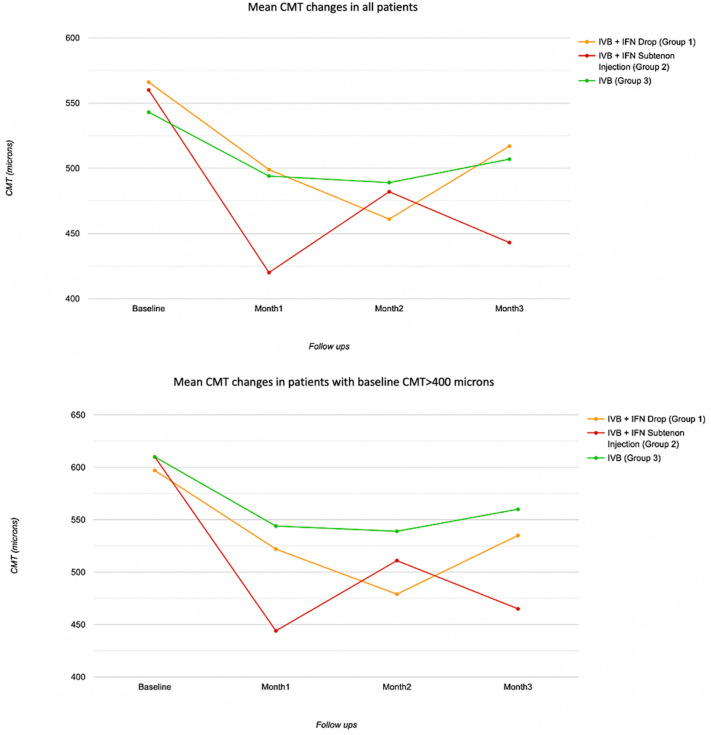
Mean central macular thickness (CMT) change in all three groups of patients in follow up visits (upper linear graphs) and also in patients with baseline CMT > 400 microns (lower linear graphs). CMT, central macular thickness, IVB, intravitreal bevacizumab, INF, Interferon alpha 2b

In this study, 20 (86.9%), 13 (81.2%), and 15 (75%) eyes in groups 1, 2, and 3 had CMT > 400 microns, respectively (Table 3). Comparing groups based on the mean baseline, first month, second month, and third month’s CMT and CDVA in eyes with CMT > 400 microns showed no significant difference among groups (p-value > 0.05) except for the first month CMT (p-value = 0.049). In subgroup analysis, mean CMT 1 month following injection in eyes receiving IFN sub-tenon injection (Group 2) was significantly lower than eyes treated with IVB alone (Group 3) (444 ± 123 µm vs 544 ± 96 µm; p-value = 0.042). Additionally, these eyes had lower CMT at the final visit in comparison to their baseline value (465 ± 133 µm vs 610 ± 156 µm, p-value = 0.035). Alteration of CDVA was not statistically significant among groups and within groups at each follow-up time points (all p-value > 0.05) (Fig. 1).

**Table 3:** Comparing groups based on CMT and CDVA among groups at each time point in eyes with CMT > 400 microns

Variables		Groups			Total P-value	P-value (Group 1 vs. 2)	P-value (Group 1 vs. 3)	P-value (Group 2 vs. 3)
		IVB + IFN Drop (Group 1)	IVB + IFN Injection (Group 2)	IVB (Group 3)				
CMT (microns)	Baseline	597 ± 199	610 ± 237	610 ± 156	0.973^†^	0.998	0.995	0.999
	1st month	522 ± 224	444 ± 123	544 ± 96	0.049*	0.460	0.970	**0.042**
	Change	− 74 ± 167	− 166 ± 210	− 65 ± 115				
	P-within^§^	0.246	**0.018**	0.134				
	2nd month	479 ± 171	511 ± 178	539 ± 94	0.390*	0.933	0.438	0.938
	Change	− 118 ± 158	− 99 ± 111	− 71 ± 97				
	P-within^§^	**0.004**	0.140	**0.021**				
	3rd month	535 ± 223	465 ± 133	560 ± 100	0.090*	0.566	0.958	0.083
	Change	− 61 ± 182	− 145 ± 231	− 50 ± 96				
	P-within^§^	0.722	**0.035**	0.222				
CDVA (LogMAR)	Baseline CDVA	0.89 ± 052	0.75 ± 0.46	0.89 ± 0.49	0.668^†^	0.814	0.999	0.824
	1st month	0.81 ± 0.51	0.69 ± 0.28	0.77 ± 0.33	0.653*	0.785	0.989	0.876
	Change	− 0.08 ± 0.38	− 0.06 ± 0.40	− 0.12 ± 0.28				
	P-within^‡^	0.999	0.999	0.486				
	2nd month	0.68 ± 0.40	0.82 ± 0.40	0.87 ± 0.41	0.328*	0.649	0.410	0.987
	Change	− 0.21 ± 0.38	0.07 ± 0.21	− 0.02 ± 0.19				
	P-within^‡^	0.080	0.999	0.999				
	3rd month	0.71 ± 0.48	0.70 ± 0.36	0.85 ± 0.38	0.482*	0.999	0.700	0.619
	Change	− 0.18 ± 0.42	− 0.05 ± 0.26	− 0.04 ± 0.12				
	P-within^‡^	0.324	0.999	0.999				

Bold values denote statistical significance at the p value < 0.05 level

^†^Based on ANOVA, between comparison adjusted by Bonferroni method

^§^Based on linear mixed model, comparison with baseline value, adjusted for the multiple comparisons by Bonferroni method

*Based on ANCOVA, adjusted for the baseline value, between-group comparison adjusted by Bonferroni method

^‡^Based on Wilcoxon signed rank test, compared with baseline value

Two patients in group 2 (sub-tenon injection of IFN-α) had flu-like syndrome which was controlled by administering acetaminophen. However, there was no side effect in other groups.

## Discussion

The management of DME refractory to IVB represents a significant challenge in clinical practice. Although off-label use of IVB for DME is an interesting option especially in low-income countries, persistent DME has been reported to be more likely with this agent than other approved anti-VEGFs [26]. Switching to other anti-VEGF agents, application of macular laser photocoagulation, or addition of steroids are among the most common options. However, treatment response is still not optimum, as according to previous reports 42% of patients who were unresponsive to IVB after switching to aflibercept also failed to respond, and sustained steroid therapy imposes collateral adverse effects such as progression of cataract and IOP rise [27, 28].

In the present study, we evaluated the additive effect of two routes of administration of IFN-α2b (topical drop and sub-tenon injection) to continued bevacizumab therapy in patients with refractory DME to 3 consecutive IVB injections. Based on our finding, IFN causes an additional CMT reduction in comparison to Bevacizumab monotherapy, and the reduction was more prominent in the IFN sub-tenon injection group (− 117 µm in sub-tenon group at month 3 vs − 49 µm in IFN drop, and − 36 µm in Bevacizumab monotherapy); however, it can be attributed to the relatively small sample size of the study in detecting the difference in data with wide standard deviation that this difference did not reach statistical significance. Similarly, LogMAR CDVA was also lower in sub-tenon IFN injection (0.61 ± 0.37) in comparison to IFN drop (0.71 ± 0.46) and bevacizumab monotherapy (0.75 ± 0.38) and alteration of CDVA was higher in the IFN drop group. However, none of these changes were statistically significant between the groups.

Previous reports demonstrated promising results following IFN drop administration for uveitis induced cystoid macular edema and pseudophakic cystoid macular edema and supports the mechanism of stabilization of the blood-retina barrier by this agent [19, 21, 29]. In a case series that assessed the effect of topical IFN-α 2b in the treatment of refractory DME, five eyes of three patients were enrolled. Patients received IFN-α 2b drops four times a day and 1 month after the treatment, improvement in OCT findings and visual acuity in all eyes were achieved that remained stable during the 3-month follow-up. They reported conjunctival injection and follicular conjunctivitis as the side effects of topical administration that were treated with lubrication and steroids [11]. Our study, not only has the advantage of studying more cases (n = 23 comparing to n = 5), but also has the comparative design rather than reporting a series of cases.

Afarid et al. in their randomized clinical trial compared the effect of monthly intravitreal anti-VEGF injection and topical IFNα-2b drop with standard anti-VEGF monthly injection on patients with refractory DME. The patients treated by IFN drop gained higher CDVA improvement, but the difference of CMT, though lower in the IFN drop group, was not statistically significant [12]. The authors related this observation to the priority of the physiologic effect of IFN to the anatomic response. Interestingly based on our results, significant visual acuity improvement was observed only in the IFN drop group (0.91 ± 0.56 logMAR at baseline to 071 ± 0.46 logMAR at month 3, p-value = 0.010). Further research is needed to determine whether the functional effect of IFN drop dominates anatomical outcomes, as patients allocated to IFN drop had lower baseline CDVA than the other groups in our study, indicating that there is possibility for significant change.

To the best of our knowledge, there is only a case report that assessed the effect of sub-tenon injection of IFN-α2a in refractory DME. Cellini et al. reported a 66-year-old patient with DME refractory to macular laser treatment and intravitreal injection of Triamcinolone who was treated with a cycle of three sub-tenon injections/week of IFN-α2a. The patient reported a significant improvement in CDVA (from 20/200 to 20/40) and a significant reduction in CMT (from 498 to 237 µm) after 1 year of follow-up [25]. In another study, they also showed the safety and effectiveness of sub-tenon injection of 1mIU/ml interferon-α in 20 patients with refractory neovascular age related macular degeneration [24].

According to our results, patients who received sub-tenon IFN injection had lower CMT at their final visit in comparison to the baseline value. Although these eyes had also better CDVA, and lower CMT, these differences were not statistically significant in comparison to other groups. Additionally, we evaluated patients with baseline CMT > 400 µm and found that those eyes treated with sub-tenon IFN injection have lower CMT at 1 month in comparison to monotherapy with bevacizumab (444 ± 123 µm vs 544 ± 96 µm, p-value = 0.042). It is plausible that patients with higher baseline CMT could gain a higher reduction in thickness, which makes it conceivable that the additive effect of IFN injection to be noticed. However, the effect of sub-tenon injection did not sustain to the final follow up, which might be consistent with the methodology of this study, since we only injected once at baseline and prominent CMT reduction was observed after 1 month.

The half-life and clearance of sub-tenon IFN can be linked to the rapid and transitory reduction of CMT after sub-tenon injection of IFN, which did not last more than 1 month. Previous studies have shown that the duration of action for sub-tenon triamcinolone is usually 2–3 months, and the location serves as a depot for the steroid slow release throughout that time [30, 31]. But, to the best of our knowledge, there is no study to assess the duration of action in sub-tenon IFN injection. The pathophysiology behind anti-VEGF resistant leakage is attributed to alternative proangiogenic pathways mediated by platelet-derived growth factor, fibroblast growth factor, placental growth factor, interleukins, and transforming growth factor-β. The possible additive effect of IFN therapy in DME may root back to its anti-angiogenic and anti-inflammatory effects other than the VEGF pathway. As it is demonstrated in tumor models, the potent anti-angiogenic mechanism of IFN could be exerted through inhibition of these alternative pathways such as inhibition of fibroblast growth factor and IL-8 [32, 33]. Interestingly, the aqueous level of IL8 was higher in patients with DME who were unresponsive to intravitreal Bevacizumab [34].

Additionally, sustained suppression of VEGF seems also crucial in treatment of patients with refractory DME. Ferris et al. investigated data of 2 multicenter clinical trials (CATT and DRCR.net) and evaluated the patients who met the criteria for switching to another anti-VEGF agent but were continued on receiving their original assigned treatment. They showed visual acuity improvement and CMT reduction after 3 months in these patients [35]. Similarly, based on our results, IVB monotherapy and IVB plus IFN drop groups demonstrated significant anatomic improvement at 2 months following enrollment. However, despite being lower than baseline, CMT in the third month was not statistically different from baseline values, which is not surprising given the refractory nature of these eyes. As a result, without a comparison group, it’s impossible to say whether any improvement shown after adding a drug like interferon was due to the new therapy or to the mean and time regression effects seen in the control group.

Of course, this study has some limitations. The participants were not randomly allocated to treatment groups, which may expose the results to selection bias. However, eligible patients were divided into three groups blindly based on numerical order and baseline characteristics of the participants were not different. An ophthalmologist who was unaware of the treatment groups followed the patients and analysis of the results and images were performed by a masked observer. We had a relatively small sample size which may mask the true effect of treatment, therefore an RCT with an adequate number of cases is recommended. Additionally, we did not continue injection of sub-tenon IFN after month one, and we aimed to check out the additive therapeutic effect of a single sub-tenon INF injection on IVB injections.

The follow up period was set in line with previous studies for 3 months, however, long term outcomes should be studies. [11, 12] Both eyes of some patients included in the current study. This might be a source of bias because there could have been some systemic impacts. It should be noted, however, that in patients who had more than one eye included, both eyes underwent the identical procedure. Finally, we did not evaluate other aspects of vision such as contrast sensitivity which is also impaired in patients with DME and the alteration could be achieved after treatment with the same level of CDVA.

## Conclusion

In our prospective study, we found that patients with refractory DME who had sub-tenon IFN injection at the time of enrolment had a significant CMT reduction, particularly when the baseline CMT was greater than 400. Although the controversy persists, based on our findings and the pathophysiologic rationale of the action of IFN, randomized trials with greater sample sizes and testing additional methods of administration, such as monthly sub-tenon injection of IFN, are recommended.

## Data Availability

The datasets used in the current study are available upon reasonable request.

## Abbreviations

IFN: Interferon
IVB: Intravitreal Bevacizumab
Anti-VEGF: Anti-vascular endothelial growth factor
CDVA: Corrected distant visual acuity
CMT: Central macular thickness
DME: Diabetic macular edema
CI-DME: Center-involved DME
NSAIDs: Nonsteroidal anti-inflammatory drugs
OSSN: Ocular surface squamous neoplasia
IOP: Intraocular pressure
RCT: Randomized clinical trial
